# Improved Efficiency of Pomegranate Seed Oil Administrated Nasally

**DOI:** 10.3390/pharmaceutics14050918

**Published:** 2022-04-22

**Authors:** Hiba Natsheh, Elka Touitou

**Affiliations:** The Institute for Drug Research, School of Pharmacy, Faculty of Medicine, The Hebrew University of Jerusalem, Jerusalem 9112102, Israel; hiba.natsheh@mail.huji.ac.il

**Keywords:** pomegranate seed oil, nasal delivery system, memory impairment, movement impairment, delivery to brain, improved memory, phospholipid oily gel

## Abstract

Pomegranate seed oil (PSO) is currently administrated orally as a food supplement for improving memory. However, the efficiency of the oral dosage forms for such purposes is low, mainly due to the blood brain barrier impeding a good delivery to brain. In this work, we designed and characterized a PSO phospholipid oily gel for nasal administration. We tested the performance of the new PSO delivery system in animal models for impaired memory and locomotor activity. The experimental results indicated a statistically significant improvement (*p* < 0.05) of more than 1.5 fold in the behavior of animals treated nasally, in comparison to those treated with orally administrated oil. Furthermore, in multiphoton microscopy and near infrared imaging studies, the nasal administration of fluorescent probes, fluorescein isothiocyanate (FITC), and indocyanine green (ICG) incorporated in the PSO system showed enhanced delivery to the brain. Results of the histopathologic examination of the nasal cavity and mucosa, as carried out by a pathologist, indicated the safety of the PSO phospholipid oily gel. In conclusion, the results of this work encourage further investigation of the phospholipid oily gel composition as a new way of PSO administration.

## 1. Introduction

The plant *Punica granatum* (*Punicaceae* family) is known for its medical uses since ancient times. Pomegranate seed oil (PSO) contains polyunsaturated fatty acids, monounsaturated fatty acids, and saturated fatty acids. PSO exhibits in vitro antioxidant, anti-inflammatory, and neuroprotective activities [1,2,3]. This oil is currently administrated orally for memory improvement and prevention of the progression of neurodegenerative diseases [4,5,6]. 

Growing evidence suggests that pomegranate seed oil and extract have therapeutic potential for the treatment and prevention of various central nervous system diseases. Sarkaki reported that two weeks of oral administration of pomegranate seed extract improved the memory of rats after ischemic injury and permanent cerebral ischemia [7,8]. Other studies have focused on the neuroprotective effect of long-term oral administration of PSO nanoemulsions for the prevention and treatment of neurodegenerative disease in an animal model of genetic Creutzfeldt–Jacob disease (CJD) [5]. Braidy et al. [9] demonstrated evidence of protection against the loss of synaptic structure proteins and neuro-inflammation in a transgenic mice model of Alzheimer’s disease (AD) that consumed a diet containing 4% ground pomegranates for 15 months.

However, the oral administration of PSO results in partial bioavailability due to its high metabolism in the GI [10]. Moreover, the efficiency of the oral dosage forms for the management of brain disorders is low. This is mainly due to the blood brain barrier (BBB) that limits the absorption of molecules into the brain [11].

The nasal pathway is an alternative route of administration of active substances. It has been traditionally used to treat localized ailments such as allergic rhinitis and symptoms of common cold. For more than three decades, this route has been explored for the systemic delivery of drugs [12]. More recently, the possibility of nasal drug delivery to the brain and the central nervous system (CNS) has become an attractive field of research [13,14,15,16]. The main advantages of the nasal pathway reside in the possibility to bypass the BBB and to avoid the hepatic first-pass metabolism associated with the oral intake [12,17].

Despite the advantages of this route, nasal administration of pure PSO can be inconvenient due to nasal leakage or aspiration into the lungs [18]. Furthermore, the low permeation across the nasal mucosa prevents good absorption of many molecules [12].

Therefore, there is a need for new approaches to overcome the present obstacles for the nasal administration of PSO. Many strategies were investigated to enhance the nasal delivery to brain of various molecules. These include nanoparticles and surfactants. Nanovesicular carriers have been shown to be very efficient for the treatment of multiple sclerosis [13], hot flushes [14], pain [15,16,19,20,21], Parkinson’s disease [19], inflammation, migraine, and insomnia [22,23].

In this work, we investigated the efficiency of the nasal delivery of a PSO phospholipid oily gel in animal models for impaired memory and movement. To test the treatment efficiency of the nasal administration of PSO systems, we used mouse models of memory and movement impairment and compared the effect to that of oral administration of the pure oil. The ability to deliver to the mouse brain was tested using fluorescent probes in multiphoton microscopy and near infrared (NIR) imaging studies. The potential irritation of the nasal PSO system on the nasal cavity was examined following sub-chronic administration of the composition to rats.

## 2. Materials and Methods

### 2.1. Materials

PSO, containing (*w*/*v*) 90% polyunsaturated fatty acids, 5% monounsaturated fatty acids and 5% saturated fatty acids (based on the certificate of analysis provided by N.S. Oils Ltd., Kibbutz Saad, Israel) was used in this study. The phospholipids (PL) Phospholipon 90G, Lipoid, Germany, and Lecithin soya, Fagron, Spain, were used in this work. Reserpine, Fluorescein isothiocyanate (FITC), Indocyanine green (ICG), Sesame oil, and Tween 80 were purchased from Sigma Aldrich, Israel. Dimethyl sulfoxide (DMSO) was purchased from Bio Lab Ltd., Jerusalem, Israel. Propylene glycol (PG) and vitamin E were bought from Tamar, Israel. *Olivae Oleum* (Oleic acid) from Henry Lamotte Oils GMBH, Bremen, Germany was used. All other chemicals were of pharmaceutical grade.

### 2.2. Animals

All procedures carried out on animals were according to the National Institute of Health’s regulations and were approved by the Committee for Animal Care and Experimental Use of the Hebrew University of Jerusalem (MD-11-12821-3; MD-19-15895-3; MD-21-16402-3; MD-21-16474-4).

Mice (male CD-1 ICR) 24–33 g and rats (male HSD) 220–250 g were housed under standard conditions of light and temperature in plastic cages in the specific-pathogen unit (SPF) of the School of Pharmacy at the Hebrew University of Jerusalem. Animals were kept in separated cages with smooth flat floors and provided with unlimited access to water and food.

Nasal administration of the PSO delivery systems was performed under short (2–3 min) anesthesia with isoflurane to prevent loss of the formulation by sneezing. The animals were held in an upright position to mimic human position. The compositions were applied to both nostrils of the animal by a positive displacement pipette coupled with CP25 capillaries and pistons (Microman^®^, precision microliter pipette, Gilson, France).

### 2.3. Methods

#### 2.3.1. Preparation of PSO Phospholipid Nasal Oily Gel Systems

The systems comprised of PSO and PL at a weight ratio of 2:1–4:1. The two components were mixed using a mortar and pestle at room temperature until a homogenous gel was obtained. Then, vitamin E (0.5% *w*/*w*) was added as an antioxidant. Then, to the obtained gel, other additives such as vegetable oils (oleic acid and sesame oil) up to 40% *w*/*w*, and/or propylene glycol (20–75% *w*/*w*) could be added. The additives were gradually mixed with the gel using an overhead stirrer (Heidolph, Hei Torque 200, Schwabach, Germany) at 500 rpm for 5 min.

Four PSO delivery systems containing 2:1, 2.3:1, 3:1, or 4:1 PSO:PL ratios were prepared.

#### 2.3.2. Characterization of PSO Phospholipid Nasal Oily Gel Systems

Nasal systems containing 2:1, 2.3:1, 3:1, or 4:1 PSO:PL ratios were characterized for their appearance, spreadability, viscosity, and pH.

The spreadability of the PSO systems were measured by a modified method published by Sherafudeen and Vasantha [24]. This parameter was determined using a 7.6 × 2.6 cm^2^ glass slide (Marienfield, Lauda-Konigshofen, Germany). The basal side of porcine skin (Kibuttz Lahav, Negev, Israel) was tied to the surface of the slide with a thread. The slide was kept in a mini-incubator (Labnet International, Edison, NJ, USA) at 37 °C. One hundred microliters of the PSO delivery systems were placed on the tissue at an angle of 120°. The spreadability of the system was considered to be the distance that it traveled in 10 s. The average of three readings was recorded for each system.

Viscosity measurement for PSO phospholipid nasal oily systems was carried out using a Brookfield DV III Rheometer LV (Brookfield Engineering Labs., Stoughton, MA, USA) coupled with spindle S4 at 10 rpm and room temperature.

It was interesting to know which pH is generated following the application of the formulation into the nasal cavity. For this purpose, the oily gel system was dispersed in normal saline. The pH of the dispersed system, at a volume ratio of 1:10 [24], was assessed with a Seven Easy pH meter and an InLab Expert Pro electrode (Mettler Toledo, Changzhou, China). All measurements were duplicated.

#### 2.3.3. Visualization of Nasal Delivery to Brain

##### Nasal Delivery of Fluorescein Isothiocyanate (FITC) to Mice Brain: A Multiphoton Imaging Study

In this experiment, we tested the formulation containing a ratio of PSO:PL. The ability of the PSO phospholipid nasal oily system to deliver FITC to mice brain was assessed by multiphoton imaging. A PSO system containing 0.5% FITC and 20% PSO was investigated. Mice received either 10 µL of nasal PSO system or 10 µL of a control composition containing 0.5% FITC and 20% PSO in paraffin oil. Ten minutes after treatment, the animals were sacrificed and the brains were removed and washed with normal saline. The olfactory region in the brain was observed under a multiphoton microscope A1-MP (NIKON, Tokyo, Japan). The field of image was 818 × 818 × 200 nm (width × height × depth), the scanning was performed using × 20 objective lens with an excitation wavelength of 740 nm, a laser intensity of 6%, and a scan speed of 0.125. The FITC fluorescence intensity (Arbitrary units, A.U.) in the scanned brain region was further analyzed using ImageJ software. The brain of an untreated mouse was examined to rule out the auto-fluorescence of the brain olfactory region.

##### Visualization of Nasal Delivery of Indocyanine Green (ICG) to Mice Brain: A Near Infrared (NIR) Imaging Study

The delivery of ICG to the mice brain from the nasal PSO delivery system containing PSO: PL ratio of 4:1 was examined by the Odyssey^®^ Infrared Imaging System (LI-COR, Lincoln, NE, USA).

Mice were treated with 10 µL of PSO phospholipid nasal gel comprising 0.5% ICG and 5% PSO or with a control nasal composition containing the same concentration of ICG dissolved in 99.5% PG and compared to untreated mice. Thirty minutes after treatment, the animals were sacrificed and brains were removed, washed with normal saline, and observed under the imaging system. The scanning was performed using offset 2, resolution 339.6 µm, channel 800 nm, and intensity 1. The fluorescence intensity of the probe (A.U.) in the brain was further analyzed using ImageJ software.

#### 2.3.4. Measurement of the Effect of PSO Phospholipid Nasal System in Animal Model of Impaired Memory by the Novel Object Recognition Test (NORT)

A PSO system comprised of 25% PSO at a PSO:PL ratio of 3:1 was tested in this experiment and compared to the oral administration of pure PSO. A modified reserpine induced memory impairment mice model was used [25].

The reserpinized mice were divided randomly into three groups: two PSO treated animal groups, PSO administrated nasally in the delivery system, PSO given orally, and one untreated reserpinized control group (*n* = 6/group). An additional untreated and un-reserpinized group served as a control with normal memory to test the suitability of the animal model. Reserpinized mice in the treatment groups received PSO at a dose of 300 mg/kg twice daily for five days and the last dose was given on the sixth day, one hour before running the behavioral test.

On days three and five of the experiment, the animals of the three reserpinized groups received intraperitoneal injections of reserpine at doses of 0.5 and 0.4 mg/kg from 0.05% and 0.004% reserpine solutions, respectively, at an injection volume of 10 mL/kg. The reserpine injections were prepared by suspending the compound in water for injection containing 0.1% DMSO and 0.3% Tween 80.

A modified NORT test [25,26] was conducted in a transparent individual cage (29 × 28.5 × 30 cm) for each mouse. Briefly, the animals were placed in the cage to explore two objects similar in terms of size, shape, and color for 6 min. Then, they were returned to their home cage and the arena, including the objects, was thoroughly cleaned with 70% alcohol to avoid leaving odorous cues. After one hour, one of the familiar objects was replaced with a novel object (different in size, shape, and color). The mice were returned to the testing arena and their ability to explore each object was recorded for five minutes. Exploration was defined as the orientation of an animal’s snout toward the object, sniffing, or touching. The percentage of novel object recognition was calculated as follows: the time spent exploring the new object × 100/time total of exploration (time exploring new object + time exploring familiar object).

#### 2.3.5. Evaluation of Motor Behavior of Reserpinized Mice in Open Field Test: Effect of PSO Phospholipid Delivery System Administrated Nasally vs. Oral Administration of the Oil

The goal of this experiment was to evaluate the effect of PSO administrated nasally in the phospholipid oily gel carrier in comparison to orally administrated PSO. A PSO system comprised of 25% PSO at a PSO:PL ratio of 3:1 was tested. The motor behavior of reserpinized mice modified model of movement impairment was tested using the open field test [27].

The reserpinized mice were divided randomly into three groups: two PSO treated groups, the PSO nasal system and the PSO oral system, and one untreated control group (*n* = 6/group). Animals in the treatment groups received PSO at a dose of 300 mg/kg twice daily for five days, then the last dose was given on the sixth day 1.5 h before the test. On the fifth day of the experiment, the animals of the three groups received an intraperitoneal injection of 4 mg/kg Reserpine.

The spontaneous locomotor activity of the animals was measured 23 h after the reserpine injection and 1.5 h after the last administration of PSO. Mice were placed in the center of a cage (29 × 28.5 × 30 cm) with a flat floor divided into nine equal squares. The number of squares crossed by the animal was counted for 5 min, without a habituation session.

#### 2.3.6. Assessment of Local Safety of PSO Phospholipid Oily Gel on Rats’ Nasal Cavity and Mucosa following Sub-Chronic Administration

In this test, the safety of the PSO nasal system containing 25% PSO at a PSO:PL ratio of 3:1 on the nasal cavity and mucosa was evaluated in rats using a method previously described by Duchi et al. [20]. In brief, nine male HSD rats (220–250 g) were divided equally into two treatment groups and one untreated control group. Rats in the treatment groups received nasally 15 μL PSO phospholipid oily system or normal saline into both nostrils twice a day, for one week. At the end of the experiment, the animals were sacrificed and the nasal cavities were removed and fixed in 3.7% formaldehyde PBS. Sections of the nasal cavity were cut serially at 5 μm thickness and stained with hematoxylin and eosin. The sections were examined by a histopathologist (Authority for Animal Facilities, Hebrew University of Jerusalem, Jerusalem, Israel) using an Olympus light microscope BX43 and an Olympus digital camera DP21 with Olympus cellSens Entry 1.13 software (Olympus, Tokyo, Japan) using a magnification of ×10. Local toxicity was assessed by evaluating the histopathological alterations in different regions of the nasal cavity including cartilage and turbinate bone, lamina propria and submucosa, mucosal epithelium, and lumen.

#### 2.3.7. Statistical Analysis

Data are reported as mean ± SD and analyzed using one-way ANOVA with Bonferroni’s multiple comparison test or using a two-tailed Mann–Whitney test using the GraphPad Prism 5 software (GraphPad Software, Inc., San Diego, CA, USA). A *p* < 0.05 is considered significant in all cases.

## 3. Results

### 3.1. Properties of PSO Nasal Phospholipid Oily Gel Systems

PSO phospholipid systems composed of PSO:PL at various ratios were characterized by their appearance, viscosity, spreadability, and pH. Their properties are presented in Table 1.

PSO phospholipid oily gel systems are homogenous low viscosity gels. The viscosity of these PSO systems was found to be higher than that of the pure oil which is liquid at RT with a viscosity value of 21.5 ± 1.06 cP. The viscosity of the nasal delivery systems increased proportionally with the PL ratio, ranging between 72 cP for the system containing 4:1 PSO: PL and 488 cP for the system containing the two components at a ratio of 2:1. It is noteworthy that a higher viscosity is advantageous in nasal administration, avoiding oil leakage and lung aspiration. The range of spreadability of the formulations was 0.87–1.17 cm. The obtained pH range of 5.71–6.38 is considered acceptable for nasal administration.

### 3.2. Nasal Delivery to Brain from the PSO System: A Multiphoton Microscopy Study

**Figure 1:**
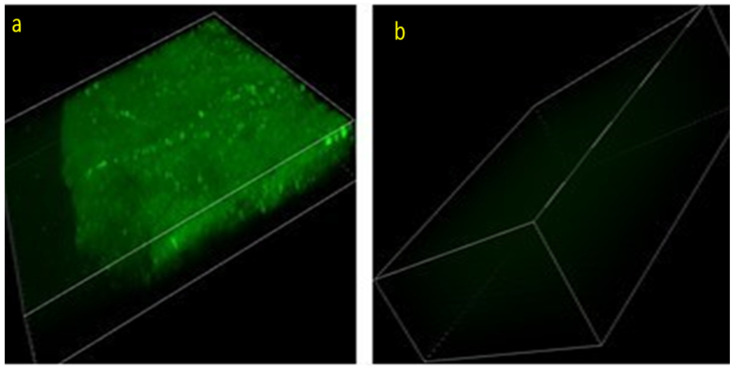
Representative multiphoton micrographs for the olfactory region of mouse brains treated nasally with (**a**) PSO nasal phospholipid system or (**b**) control composition containing PSO but lacking PL, each containing 0.5% *w*/*w* FITC. Field of images: Height = 818 µm and width = 818 µm for the both images. Depth = 100 µm for brains of mice which received PSO system and 200 µm for those who received the control composition. Lens × 20 (A1-MP microscope NIKON—Tokyo, Japan).

Figure 1 presents multiphoton micrographs for the olfactory region of mouse brains 10 min after nasal treatment with FITC in the PSO phospholipid delivery system versus a control containing the same PSO concentration but without PL.

It can be seen that the nasal administration of PSO phospholipid oily gel containing 0.5% FITC yielded a strong fluorescent signal in the olfactory region of the animal brain as compared to the control FITC composition.

Semi-quantification of the obtained brain images gave a fluorescent intensity of 51.7 A.U. for the PSO Nasal system. On the other hand, nasal administration of FITC from a composition containing an equal amount of PSO not in the gel system yielded a fluorescent intensity of only 9.4 A.U. The multiphoton micrographs and their semi-quantification indicated a more than 500% increased delivery.

### 3.3. Delivery to the Brain of Molecules from the Nasal Phospholipid Gel System: An NIR Imaging Study

**Figure 2:**
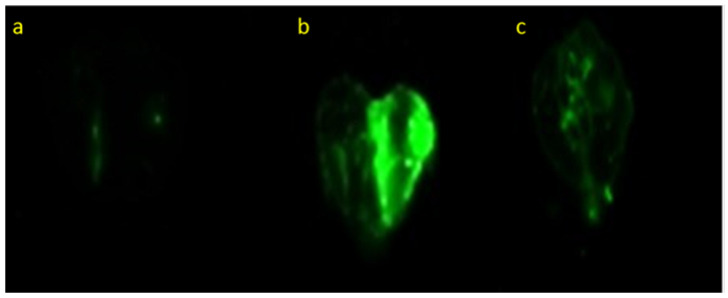
Representative NIR images for (**a**) brain of untreated mice (**b**) brain of mice 30 min following nasal treatment with the PSO delivery system and (**c**) brain of mice treated with a control composition. Each system contained 0.5% *w*/*w* ICG. Odyssey^®^ Infrared Imaging System (LI-COR, Lincoln, NE, USA).

In this experiment, the effect of the PSO phospholipid system on the delivery of ICG to brain was examined in mice by NIR imaging of the brain 30 min following treatment. The obtained NIR images show that the nasal administration of the PSO phospholipid oily gel system yielded a strong fluorescent signal of ICG, as compared to the control composition of the probe (Figure 2).

The semi-quantification of the images and the normalization of the fluorescence intensity (by subtracting the auto-fluorescence of the untreated brain from the fluorescence intensity of each image) showed a fluorescent intensity of 7.5 A.U. for the nasal PSO phospholipid system. On the other hand, nasal administration of the control ICG composition yielded a fluorescent intensity of only 0.4 A.U. This result points towards the role of the PSO phospholipid oily gel nasal system in improving the delivery of this molecule to the brain.

### 3.4. Effect on Memory of an Animal Model

#### 3.4.1. Animal Model of Memory Impairment

**Figure 3:**
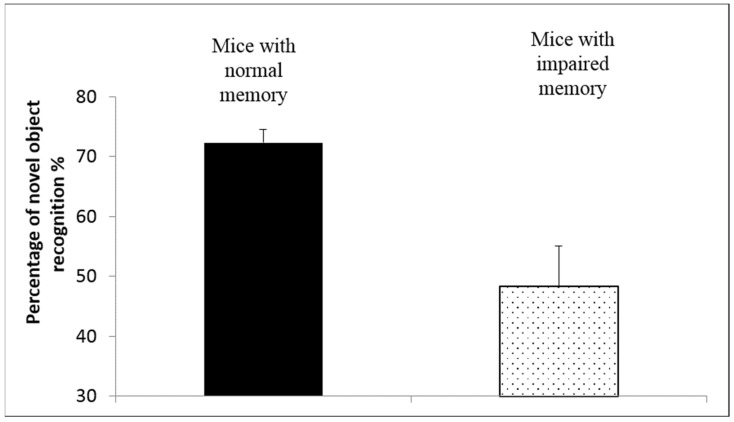
Percentage of novel object recognition for mice with normal memory and for mice with impaired memory (*n* = 6/group), (mean ± SD). *p* < 0.001 for mice with normal memory vs. mice with impaired memory, determined by two-tailed Mann–Whitney Test.

The reserpine-induced memory impairment mice model and the novel object recognition test were used to evaluate the effect of PSO phospholipid nasal oily gel on memory. The first step was to validate the reserpine-induced memory impairment animal model by comparing the behavior of untreated reserpinized animals to those of normal memory. The results indicated that animals with normal memory exhibited a recognition percentage of 72.3 ± 2.2%, as compared to 48.3 ± 6.8% for animals with impaired memory (*p* < 0.001) (Figure 3). This 60% reduction in the animal’s memory following reserpine sub-chronic administration indicates the suitability and reliability of the impaired memory model.

#### 3.4.2. Effect of Nasal Administration of PSO Phospholipid Oily System on Animal Behavior in the Animal Model for Impaired Memory

The effect of the PSO phospholipid nasal system on the behavior of animals with reserpine-induced memory impairment was evaluated in this experiment in comparison to the oral administration of PSO. The data obtained in this part of the experiment showed that pretreatment and treatment with the PSO nasal system for six days led to a significant increase in the recognition percentage in mice with impaired memory.

**Figure 4:**
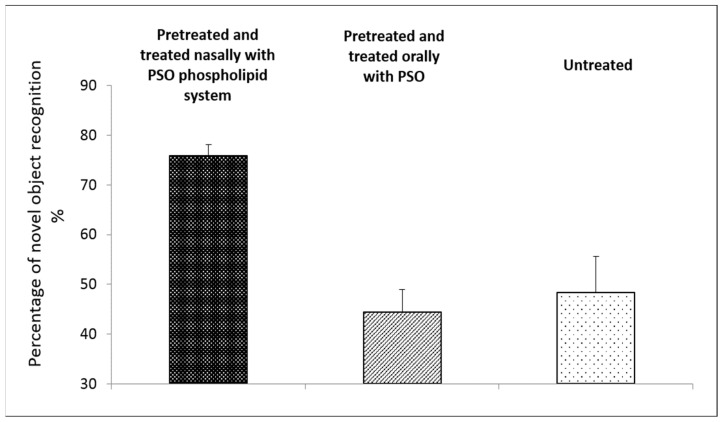
Percentage of novel object recognition by three groups of mice with impaired memory: 1. Pretreated and treated twice daily nasally for six days with the PSO oily phospholipid gel, 2. pretreated and treated twice daily for six days orally with PSO, and 3. untreated animals (*n* = 6/group), (mean ± SD). *p* < 0.001 for nasal PSO nasal system vs. untreated group with impaired memory and *p* < 0.001 for nasal PSO nasal system vs. orally treated, as determined by one-way ANOVA with Bonferroni’s multiple comparison test.

Figure 4 shows a novel object recognition percentage of 75.9 ± 4.5% in mice pretreated and treated with the PSO phospholipid oily gel. On the other hand, the recognition of animals treated with PSO orally was only 44.4 ± 7.4%, similar to the untreated mice with impaired memory (*p* > 0.05).

This superior effect of the PSO nasal system emphasizes the important role of administrating PSO nasally from the phospholipid oily gel carrier for enhanced efficiency of PSO treatment.

### 3.5. Effect of Nasal Administration of the PSO Nasal System Compared to the Oral Administration of PSO on the Motor Behavior of Reserpinized Mice: Results of Open Field Test

**Figure 5:**
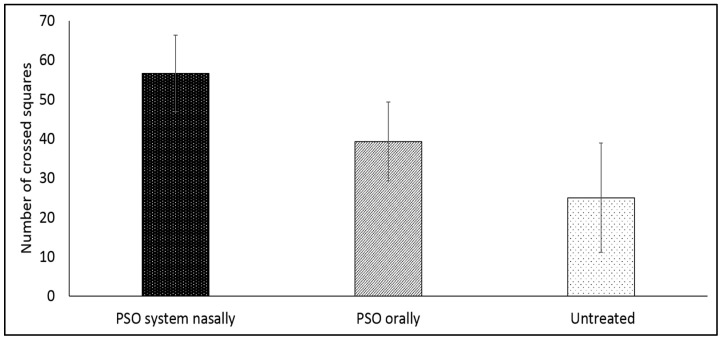
Number of squares crossed in the open field test by reserpinized mice: 1. treated twice daily for five days with PSO system nasally (PSO phospholipid oily gel nasal system), 2. treated twice daily for five days with PSO orally, and 3. untreated animals (*n* = 6/group) (mean ± SD). *p* < 0.001 for PSO nasal system vs. untreated control, *p* < 0.05 for PSO nasal system vs. PSO orally, *p* > 0.05 (considered not significant), for PSO orally vs. untreated control, as determined by one-way ANOVA with Bonferroni’s multiple comparison test.

In this experiment, animals with locomotor impairment were treated nasally with PSO phospholipid oily gel or orally with pure oil. Figure 5 presents the number of squares crossed in the open field test by 3 groups of reserpinized mice: 1. treated with PSO phospholipid oily nasal gel system, 2. treated with PSO orally, and 3. untreated animals.

Figure 5 shows that mice treated with the new PSO phospholipid oily gel nasal system expressed increased locomotor activity, crossing 56.6 ± 9.7 squares while the PSO oral group crossed only 39.3 ± 10.0 squares. The untreated animals were able to cross only 25.0 ± 13.9 squares. The results are statistically significant: *p* < 0.05 for the PSO phospholipid oily gel nasal system versus PSO orally and *p* < 0.001 for the PSO phospholipid oily gel nasal system versus the untreated control. This 200% improved movement emphasizes the effect of the new nasal PSO system to reverse the effects of reserpine and enhance locomotor activity in the tested animal model.

### 3.6. Local Safety of the PSO Phospholipid Nasal Delivery System

The safety of the PSO phospholipid oily gel system on the nasal cavity and mucosa of rats was tested following sub-chronic treatment twice daily for 7 days as compared to normal saline application or to the cavity of untreated animals.

**Figure 6:**
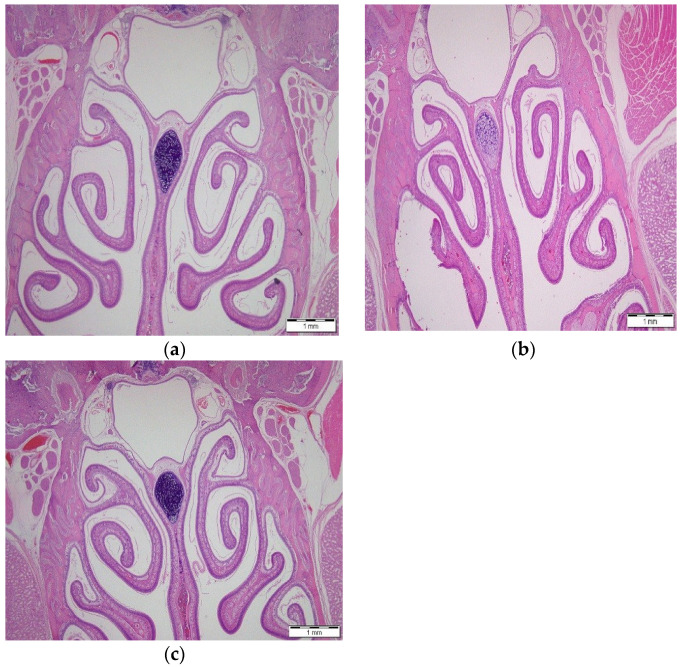
Representative micrographs of nasal cavities excised from rats: (**a**) treated nasally with PSO phospholipid gel system, (**b**) treated nasally with normal saline, and (**c**) untreated rats. Bar = 1 mm.

The histopathologic examination, carried out by a pathologist, indicates that the nasal cavity and mucosa of animals treated with the PSO phospholipid oily gel system were similar to those treated with normal saline nasally at the same treatment regimen and were also similar to untreated rats. The bone was symmetrical and the turbinate was intact. No leukocyte infiltration or loss of cilia was noticed in the lamina propria. The submucosal integrity was preserved without any loss of cilia. No evidence was found for leukocyte or non-cellular material infiltration in the lumen (Figure 6).

## 4. Discussion

This work presents a new PSO phospholipid gel delivery system for nasal administration for the treatment of impaired memory. The viscosity of the gel, as a result of the presence of phospholipid, is an important property to avoid the nasal leakage of PSO and its lung aspiration. The gel system has a relatively low viscosity (72–488 cP). Although we have not tested the residence time, another work on systems with much higher viscosity showed an improved residential time in the nasal mucosa with an increase in viscosity [28]. Another important property of PSO systems is that their pH values upon dispersion in a physiological fluid.

The novel delivery system presented in this work shows an enhanced nasal delivery of molecules to the brain and an improved efficiency of the oil tested in animals. This feature was demonstrated by multiphoton microscopy, where a five-fold increase in the delivery of FITC to the olfactory region of mouse brain ten minutes post nasal administration was evidenced, as compared to control composition. Moreover, in a NIR imaging study, nasal delivery to mice brain of ICG in PSO system, 30 min post-administration, was enhanced by 18-fold, versus a control composition. The enhanced delivery to brain of FITC and ICG, administrated nasally in the PSO phospholipid gel, emphasizes the role of the carrier. The phospholipid has two roles in this delivery carrier: it acts as a permeation enhancer and contributes to the viscosity of the gel.

The next set of experiments in this study focused on the evaluation of the effect of PSO phospholipid oily gel administrated nasally on the memory and movement of animals. Six days of treatment with the new system prevented memory impairment in the reserpinized mouse model. This was not obtained with the PSO administrated orally. Furthermore, a 200% improvement in movement behavior was evidenced in mice model of locomotor impairment, following five days of nasal administration of the PSO phospholipid system. These data point towards a stronger neuroprotective effect of PSO when administrated nasally in the phospholipid gel but not following oral administration of PSO.

The neuroprotective mechanism of PSO in the brain remains unclear. It is suggested that PSO acts as a strong antioxidant against oxidative stress and free radical damage. This hypothesis is supported by an in vitro study by Sabahi et al. [29] indicating the ability of PSO to neutralize reactive oxygen species (ROS) and enhance the expression of the antioxidant gene in rat pheochromocytoma (PC12) neuronal cells neuro-intoxicated by 3-nitropropionic acid (3-NP).

Oxidative stress and free radical damage seem to play a key role in neurodegenerative disorders such as Alzheimer’s disease and Parkinson’s disease. Previous studies indicated that oxidative stress induces neuroinflammation, protein aggregation, and mitochondrial dysfunction leading to neuronal death, depression-like behavior, and memory impairment [30,31].

As mentioned in the introduction, several works have investigated the neuroprotective effect of PSO animal models of CNS diseases [5,6,7,8,9].

Recently, Fathy et al. [32] studied the effect of pomegranate seed extract on a paraquat-induced mouse model of Parkinson’s disease. Animals started to receive the extract orally on daily basis for two weeks then continued concomitantly with intraperitoneal injections of paraquat twice daily for three weeks. The reported results indicated that the treatment reduced oxidative stress via a significant reduction of malondialdehyde levels and augmentation of antioxidant enzyme activity in the substantia nigra region of the brain.

Previous publications investigated the potential therapeutic effect of long-term administration of pomegranate seed oil and extract. Treatment regimens in these reports reached more than 6 months [5,9]. One noteworthy finding of our work is the efficiency of PSO delivered nasally from the new system evidenced in just a few days of treatment. To the best of our knowledge, this is the first work investigating the effect of nasal delivery of PSO for the treatment and prevention of CNS diseases in a short period of time.

Nasal drug delivery is a convenient and noninvasive alternative route to the oral way of administration. By using nasal treatment, the disadvantages related to the gastrointestinal tract and hepatic first-pass metabolism can be avoided. Another advantage of nasal administration is the ability to deliver molecules directly to the brain by bypassing the BBB via the olfactory and trigeminal nerves allowing for an improved treatment [11,17].

In our previous work, we have shown that some carriers, such as soft phospholipid nanovesicles, enhanced the direct nose to brain delivery of the incorporated drug, leading to a rapid onset of action and an improved treatment outcome [13,14,15,16,18,19,20,21]. In this work, we investigated a phospholipid oily gel system of PSO for enhanced delivery of molecules to brain and improved the efficiency of PSO treatment.

The safety of nasally administrated delivery systems is a critical parameter. Therefore, we examined the local safety on the nasal cavity and mucosa following sub-chronic nasal administration of the PSO phospholipid system to rats. The histopathologic examination of the nasal cavities indicated the absence of pathological local side effects for the tested period.

Overall, the new PSO nasal system was found to enhance the oil activity without causing nasal toxicity or irritation in the tested period.

To our knowledge, this is the first investigation on nasal delivery of PSO to the brain and CNS and its efficacy for the treatment of impaired memory. It opens the door for future research focusing on a number of directions, including the antioxidant effect of PSO components delivered nasally and the stability of PSO and the nasal system.

## 5. Conclusions

In this work, a PSO phospholipid oily gel for nasal administration was designed and investigated. The system shows an enhanced nasal delivery of molecules to brain and improved efficiency of PSO to treat memory and movement impairment in animal models. The histopathologic examination of the nasal cavities indicated the absence of pathological local side effects for the tested period.

## Figures and Tables

**Table 1:** Compositions and properties of various PSO phospholipid oily gel systems.

Composition (Weight Ratio)	Appearance	Viscosity (cP)	Spreadability (cm)	pH *
PSO: PL 4:1	Yellow, slightly viscous liquid	72.7 ± 11.45	1.17 ± 0.15	5.71 ± 0.025
PSO: PL 3:1	Yellow flowing gel	240.0 ± 2.0	1.03 ± 0.06	5.87 ± 0.02
PSO: PL 2.3:1	Yellow semisolid gel	377.0 ± 5.7	0.90 ± 0.0	6.16 ± 0.04
PSO: PL 2:1	Yellow semisolid gel	488.5 ± 13.4	0.87 ± 0.06	6.38 ± 0.14

* pH of dispersed systems in saline (1:10).
